# Influence of respiratory rate and end-expiratory pressure variation on cyclic alveolar recruitment in an experimental lung injury model

**DOI:** 10.1186/cc11147

**Published:** 2012-01-16

**Authors:** Erik K Hartmann, Stefan Boehme, Alexander Bentley, Bastian Duenges, Klaus U Klein, Amelie Elsaesser, James E Baumgardner, Matthias David, Klaus Markstaller

**Affiliations:** 1 Department of Anaesthesiology, Medical Center of the Johannes Gutenberg-University, Mainz 55131, Germany; 2 Department of Anaesthesia, General Critical Care Medicine and Pain Therapy, Medical University of Vienna, Waehringer Guertel 18-20, A-1090 Vienna, Austria; 3 Institute of Medical Biometry, Epidemiology and Informatics (IMBEI), Medical Center of the Johannes Gutenberg-University Mainz, 55131, Germany; 4 Oscillogy LLC, 131 Milmont Ave, Folsom, Philadelphia, PA, 19033-3514, USA

**Keywords:** acute lung injury, cyclic alveolar recruitment, porcine model, respiratory- dependent paO 2 oscillations, varying shunt fractions

## Abstract

**Introduction:**

Cyclic alveolar recruitment/derecruitment (R/D) is an important mechanism of ventilator-associated lung injury. In experimental models this process can be measured with high temporal resolution by detection of respiratory-dependent oscillations of the paO_2 _(ΔpaO_2_). A previous study showed that end-expiratory collapse can be prevented by an increased respiratory rate in saline-lavaged rabbits. The current study compares the effects of increased positive end-expiratory pressure (PEEP) versus an individually titrated respiratory rate (RR_ind_) on intra-tidal amplitude of Δ paO_2 _and on average paO_2 _in saline-lavaged pigs.

**Methods:**

Acute lung injury was induced by bronchoalveolar lavage in 16 anaesthetized pigs. R/D was induced and measured by a fast-responding intra-aortic probe measuring paO_2_. Ventilatory interventions (RR_ind _(n = 8) versus extrinsic PEEP (n = 8)) were applied for 30 minutes to reduce Δ paO_2_. Haemodynamics, spirometry and Δ paO_2 _were monitored and the Ventilation/Perfusion distributions were assessed by multiple inert gas elimination. The main endpoints average and Δ paO_2 _following the interventions were analysed by Mann-Whitney-U-Test and Bonferroni's correction. The secondary parameters were tested in an explorative manner.

**Results:**

Both interventions reduced Δ paO_2_. In the RR_ind _group, ΔpaO_2 _was significantly smaller (*P *< 0.001). The average paO_2 _continuously decreased following RR_ind _and was significantly higher in the PEEP group (*P *< 0.001). A sustained difference of the ventilation/perfusion distribution and shunt fractions confirms these findings. The RR_ind _application required less vasopressor administration.

**Conclusions:**

Different recruitment kinetics were found compared to previous small animal models and these differences were primarily determined by kinetics of end-expiratory collapse. In this porcine model, respiratory rate and increased PEEP were both effective in reducing the amplitude of paO_2 _oscillations. In contrast to a recent study in a small animal model, however, increased respiratory rate did not maintain end-expiratory recruitment and ultimately resulted in reduced average paO_2 _and increased shunt fraction.

## Introduction

Due to a wide variety of pulmonary and extrapulmonary aetiologies, acute lung injury (ALI) and the acute respiratory distress syndrome, as defined by the American European Consensus Conference in 1994 [1], remain major challenges in modern critical care medicine. A mortality reduction was achieved within the past 10 to 15 years and accompanied by rapid progress in assessing and understanding the underlying mechanisms. Nevertheless, mortality rates of more than 30% are still described [2,3]. Mechanical ventilation is commonly regarded as the cornerstone strategy in ALI, although various other supportive therapies are known to have a beneficial effect [4,5]. However, the optimal ventilatory setting in ALI is still controversially discussed. Moreover, an additional injury to the pre-injured lung, defined as ventilator-associated lung injury (VALI), can result from mechanical ventilation. Systemic or pulmonary inflammation caused by mediators that are released within the lung through injurious ventilation (biotrauma) is the consequence. Lung protective ventilation strategies aiming to minimize baro- and volutrauma by limitation of inspiratory pressure and tidal volume led to improved survival outcome and are accepted as the current standard therapy [6,7]. In addition to volutrauma (stretch injury), atelectrauma (cyclic alveolar recruitment and derecruitment, R/D), is another major component of VALI. Within the injured lung, inspiratory pressure can lead to an opening of collapsed lung areas, which immediately re-collapse in expiration. This breath by breath collapse is associated with shear-stress and inflammation and, therefore, promotes the pre-existing injury of lung tissue [8]. Cyclic R/D has recently been the focus of research studies, particularly in terms of clinical detection and treatment [9,10]. Variations of the pulmonary shunt fraction caused by cyclic R/D that can induce respiratory-dependent oscillations of the arterial partial pressure of oxygen (paO_2_) were described by Baumgardner *et al. *by means of ultrafast, invasive measurement of the paO_2 _[11]. The occurrence and amplitudes of paO_2 _oscillations (ΔpaO_2_) can be used to quantify the extent of cyclic R/D [10]. Theoretically, positive end-expiratory pressure (PEEP) that exceeds the lower inflection point of the static pressure-volume-curve should effectively maintain end-expiratory recruitment, minimize cyclic R/D induced biotrauma and improve gas exchange. However, limitations due to over-distension of non-dependent lung areas leading to volutrauma or circulatory depression may override these benefits [12-14]. Furthermore, PEEP does not respond to the dynamic behaviour and the distinct time constants underlying R/D of atelectasis [15,16]. Other experimental strategies to maintain end-expiratory recruitment include, shortening expiration times or an increased respiratory rate (RR) [11,17].

We hypothesised that: (1) a goal-directed RR and increased PEEP can both reduce the amplitude of intra-tidal Δ paO_2 _to less than 50 mm Hg; and (2) RR and PEEP adjusted to minimize intra-tidal Δ paO_2 _result in equivalent average lung recruitment by avoiding end-expiratory collapse. Hence, this study aims to compare the effectiveness of high extrinsic PEEP and an individually titrated RR (RR_ind_) as ventilatory interventions in a porcine ALI model designed to create a maximum of cyclic R/D. Furthermore, effects on haemodynamics, oxygenation and ventilation/perfusion ($\overset{\cdot }{\text{V}}/\overset{\cdot }{\text{Q}}$) distribution are characterised.

## Material and methods

After approval by the State and Institutional Animal Care Committee (Landesuntersuchungsamt Rheinland-Pfalz, Koblenz, Germany; approval number: 23177-07/G09-1-029), 16 juvenile pigs (German country race, weight 25 to 27 kg) were investigated in a prospective-randomised study. Two pilot experiments were conducted for set up and feasibility of the protocol.

### Anaesthesia and preparation

Following sedation by an intramuscular injection of ketamine (8 mg kg^-1^) and midazolam (0.2 mg kg^-1^) and vascular access via an ear vein, anaesthesia was induced by intravenous (i.v.) administration of propofol (4 mg kg^-1^), fentanyl (4 μg kg^-1^) and a single dose of pancuronium (0.15 mg kg^-1^) to facilitate endotracheal intubation. Anaesthesia was maintained by a continuous i.v. infusion of propofol (8-12 mg kg^-1 ^h^-1^) and fentanyl (0.1 to 0.2 mg h^-1^) for the entire experiment. After orotracheal intubation using a standard (internal diameter 7.5 mm) endotracheal tube, pressure-controlled ventilation (Ventilator: Servo 900C, Siemens, Erlangen, Germany) was initiated with a tidal volume of 10 to 12 mg kg^-1^, PEEP of 5 mbar, fraction of inspired oxygen (FiO_2_) of 0.3 to 0.4, inspiration to expiration ratio (I:E) of 1:2 and a variable RR to maintain normocapnia. A balanced saline solution was infused continuously (5 ml kg^-1 ^h^-1^). 

The following vascular catheters were placed by surgical cut-down: an arterial line and a PiCCO^® ^catheter (Pulsion Medical Systems, Munich, Germany) via the left and right femoral arteries, respectively; a central venous line via a femoral vein; an introducer for a pulmonary artery catheter (Edwards Lifescience, Irvine, CA, USA) via the right external jugular vein; an arterial introducer for ultrafast measurement of the paO_2 _via the right carotid artery. Spirometry and haemodynamic parameters were continuously recorded by a Datex S/5 unit (Datex Ohmeda GmbH, Duisburg, Germany). To ensure correct catheter positioning the typical pressure waveforms of the arterial, pulmonary arterial and central venous pressure were obtained and referenced to the mid-chest level. A Rapidlab 248 device (Bayer Healthcare, Leverkusen, Germany) was applied for arterial and mixed venous blood gas analyses. Body temperature was measured by a rectal probe and body surface warming was performed by a heating blanket system. Cardiac output (CO) was assessed by single-indicator transpulmonary thermodilution using the PiCCO^® ^system. Three repetitive i.v. injections of 10 ml cooled (< 8°C) balanced saline solution were given for each calibration.

### Ultrafast paO_2 _measurement

Through the right carotid introducer, a fiberoptic probe (FOXY-AL300; Ocean Optics, Dunedin, FL, USA) measuring paO_2 _based on oxygen-sensitive fluorescence quenching with a time resolution up to 10 Hz, was inserted into the ascending aorta. The probe was calibrated *in vitro *according to a previously reported routine [9,18]. The validity of the calibration was confirmed *in vivo *by repetitive blood gas analyses. For ultrafast data visualisation and storage, a dedicated acquisition software was used (OOI Sensors; Ocean Optics, Dunedin, FL, USA).

### Multiple Inert Gas Elimination Technique (MIGET)

The novel micropore membrane inlet mass spectrometry (MMIMS)-MIGET was used for assessment of the $\overset{\cdot }{\text{V}}/\overset{\cdot }{\text{Q}}$ distribution. An additional shunt calculation from blood gas samples was performed by a previously described and porcine-specific algorithm [19]. A normal saline solution containing the six inert gases, sulphur hexafluoride, krypton, desflurane, enflurane, diethyl ether and acetone, was prepared by a senior chemist (BD). Twenty minutes of i.v. application with an infusion rate of 6 ml minute^-1 ^was conducted for every measurement to ensure steady state conditions. Afterwards, simultaneous arterial and mixed venous blood (each 5 ml) was drawn over several breath cycles in gas-tight glass syringes and injected into the MMIMS-MIGET system. Retention of the inert gases was calculated as the ratio of arterial to mixed venous partial pressure. The $\overset{\cdot }{\text{V}}/\overset{\cdot }{\text{Q}}$ distribution was determined according to Evans and Wagner [20]. By means of the MMIMS-MIGET only the perfusion-based $\overset{\cdot }{\text{V}}/\overset{\cdot }{\text{Q}}$ as fractions of the CO were analysed. Hence, no dead space data are available. The following standard ranges were defined: shunt ($\overset{\cdot }{\text{V}}/\overset{\cdot }{\text{Q}}$< 0.005), low $\overset{\cdot }{\text{V}}/\overset{\cdot }{\text{Q}}$ (0.005 <$\overset{\cdot }{\text{V}}/\overset{\cdot }{\text{Q}}$ <0.1), normal $\overset{\cdot }{\text{V}}/\overset{\cdot }{\text{Q}}$ (0.1 <$\overset{\cdot }{\text{V}}/\overset{\cdot }{\text{Q}}$ < 10), high $\overset{\cdot }{\text{V}}/\overset{\cdot }{\text{Q}}$ (10 <$\overset{\cdot }{\text{V}}/\overset{\cdot }{\text{Q}}$ > 100).

### Lung injury model and baseline measurements

Baseline measurements were conducted in a healthy state and in ALI. The FiO_2 _was set and maintained at 1.0 before the baseline. ALI was induced via repetitive bronchoalveolar lavages: the endotracheal tube was clamped in inspiration, 30 ml kg^-1 ^of warmed balanced saline solution were instilled by gravity and afterwards immediately removed. Bronchoalveolar lavages were repeated to achieve a paO_2_/FiO_2 _ratio ≤ 300 mmHg at a PEEP of 5 mbar for 30 minutes. A fluid optimisation routine of 50 to 100 ml of hydroxyethyl starch was added, if the mean arterial pressure (MAP) persisted < 60 mmHg after ALI induction.

### Experimental protocol

Following baseline measurements in ALI the animals were randomised into two intervention groups: 1) increase of the PEEP to 10 to 15 mbar (PEEP group) and 2) individually titrated respiratory rate (RR_ind _group).

**Figure 1:**
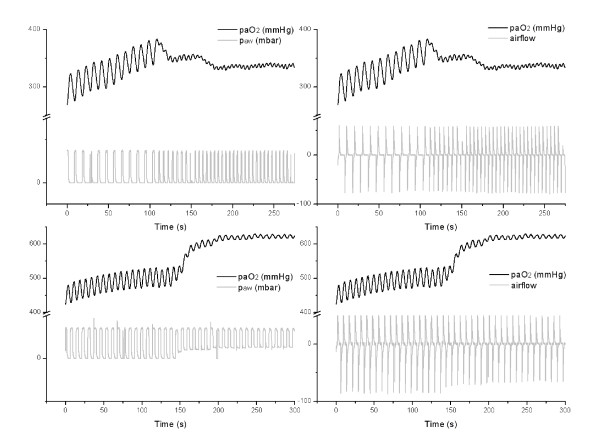
**Real-time recording of paO_2 _oscillations before and after intervention**. Exemplary real-time data (time resolution: 10 Hz) of RR_ind _(upper graphs) and PEEP (lower graphs) intervention following amplitude-stable paO_2 _oscillations representing cyclic R/D. A steady-state between recruitment and derecruitment is hardly achieved before the interventions. Correlation to airway pressure (p_aw_) and airflow demonstrates the respiratory-dependent character of Δ paO_2_.

The respirator settings were set up to peak inspiratory pressure (P_peak_) 40 mbar, zero PEEP, RR 5 to 8 minutes^-1 ^and I:E of 1:4 in pressure-controlled mode to provoke maximal cyclic R/D (time point R/D I). The inability to cause cyclic R/D with the dedicated ventilator settings was defined as obligate criterion for exclusion. ΔpaO_2 _values with a stable amplitude ≥ 50 mmHg were accepted as the pathophysiological correlate of considerable R/D in order to clearly exceed the reported variations that occur in healthy, anaesthetized animals [10,11,21]. Thereafter, the randomised intervention (Figure 1) was initiated to terminate cyclic R/D under continuous ultrafast paO_2 _measurement. In the PEEP group, extrinsic PEEP was immediately raised up to at least 10 mbar. Further increases were allowed up to a maximum of 15 mbar if Δ paO_2 _was > 20 mmHg and if the MAP was > 60 mmHg. Intrinsic PEEP was also measured dynamically as the difference between total and extrinsic set PEEP in both groups (as automated by a S/5 unit, Datex Ohmeda GmbH, Duisburg, Germany). The RR was titrated individually until a Δ paO_2 _< 20 mmHg under real-time conditions was achieved. The peak and end-inspiratory airway pressures (P_peak_, P_endinsp_) were held constant to the pre-intervention value in both groups. If haemodynamic instability, defined by a MAP < 60 mmHg, occurred after initiation of the interventions, i.v. epinephrine was administered continuously. The Δ and average paO_2 _values were reassessed 10, 20 and 30 minutes following the intervention. Spirometry and haemodynamic data were stored. Thereafter, the initial ventilator settings were set again to restore cyclic R/D (time point R/D II) and evaluate the influence of both interventions on the amount of recruitable lung tissue. MIMMS-MIGET measurements were performed during baseline and after 30 minutes of intervention. After completing the protocol the animals were euthanized in general anaesthesia by lethal injection of propofol (200 mg) and potassium chloride (40 mmol).

### Statistical methods

For a description of the continuous variables' distribution, the median and interquartile range (IQR) are given. Additional descriptive data are reported as mean and standard deviation (± SD). The intergroup difference of the main endpoints Δ and average paO_2 _following the respective intervention was analysed using the mean of the measurements over the different time points for every pig. These mean values were compared using Mann-Whitney-U-Tests. To account for multiple testing a simple Bonferroni correction was used for the two main endpoints. The multiple significance level was set at 5%. Haemodynamic data and MMIMS-MIGET derived $\overset{\cdot }{\text{V}}/\overset{\cdot }{\text{Q}}$ after 30 minutes were assessed as secondary endpoints in an explorative manner. The resulting *P*-values are descriptive only. All statistical analyses were performed using the statistical software R version 2.13.1.

## Results

A total of 16 animals were included in the study. One animal died during ALI induction and was replaced by an additional animal. In the baseline measurements before provocation of R/D, acute lung injury (paO_2_/FiO_2 _< 300 mmHg) was documented in both groups. The applied respirator settings and blood gas analyses are summarised in Table 1. 

**Table 1:** Ventilatory parameters and blood gas analyses (mean ± SD)

	PEEP group					RR_ind _group				
Parameters	Baseline ALI	R/D I	Intervention Initial	Intervention 10 to 30 minutes	R/D II	Baseline ALI	R/D I	Intervention Initial	Intervention 10 to 30 minutes	R/D II
**Ventilation**										
P_peak _(mbar)	25 ± 4	40 ± 4	40 ± 4	40 ± 4	40 ± 5	20 ± 3	38 ± 5	37 ± 3	38 ± 3	45 ± 7
P_endinsp _(mbar)	23 ± 5	35 ± 4	35 ± 4	35 ± 4	37 ± 7	19 ± 3	34 ± 4	33 ± 3	34 ± 3	40 ± 9
P_mean _(mbar)	11 ± 1	14 ± 3	21 ± 3	22 ± 3	14 ± 3	10 ± 1	13 ± 3	13 ± 3	13 ± 3	15 ± 4
RR (min^-1^)	28 ± 5	6 ± 1	6 ± 1	6 ± 1	6 ± 1	27 ± 3	6 ± 1	15 ± 2	17 ± 3	6 ± 1
PEEP_total _(mbar)	5 ± 1	0	13 ± 1	13 ± 2	0.3 ± 0.2	5 ± 1	0	0.1 ± 0.1	0.1 ± 0.2	0
V_t _(ml kg^-1^)	10 ± 1	35 ± 8	24 ± 5	24 ± 6	41 ± 9	10 ± 1	36 ± 7	32 ± 7	28 ± 6	40 ± 10
FiO_2_	1.0	1.0	1.0	1.0	1.0	1.0	1.0	1.0	1.0	1.0
T_insp _(s)	0.7 ± 0.2	2.0 ± 0.4	2.0 ± 0.4	1.9 ± 0.3	2.0 ± 0.3	0.8 ± 0.1	2.0 ± 0.3	0.9 ± 0.1	0.8 ± 0.1	2.1 ± 0.4
T_exp _(s)	1.5 ± 0.3	8.1 ± 1.8	7.9 ± 1.7	7.6 ± 1.2	8.2 ± 1.3	1.5 ± 0.3	7.9 ± 1.1	3.4 ± 0.3	3.0 ± 0.5	8.3 ± 1.4
**Blood gas analysis**										
ph	7.3 ± 0.1			7.3 ± 0.1		7.4 ± 0.1			7.5 ± 0.1	
paO_2 _(mmHg)	199 ± 69			539 ± 72		219 ± 85			163 ± 71	
paO_2_-Foxy (mmHg)	187 ± 48	307 ± 90	500 ± 86	550 ± 96	353 ± 131	223 ± 58	372 ± 111	381 ± 131	174 ± 62	209 ± 139
paCO_2 _(mmHg)	62 ± 12			57 ± 9		55 ± 3			32 ± 7	
Hb (mg dl^-1^)	8 ± 1			9 ± 1		9 ± 2			9 ± 2	
BE (mmol l^-1^)	2 ± 4			2 ± 3		3 ± 6			4 ± 4	
Q_s_/Q_t _(%)	26 ± 6			6 ± 5		24 ± 5			25 ± 6	

P_mean_, mean airway pressure; PEEP_total_, PEEP_extrinsic _+ PEEP _intrinsic_; T_insp_, inspiration time; T_exp_, expiration time; paO_2_-Foxy, paO_2 _measured by the ultrafast invasive probe. (Foxy-AL 300); Q_s_/Q_t _(%), calculated pulmonary shunt fraction. No blood gases were sampled during presence of high respiratory-dependent oscillations of the paO_2 _due to high fluctuations.

### Cyclic recruitment and oxygenation

**Figure 2:**
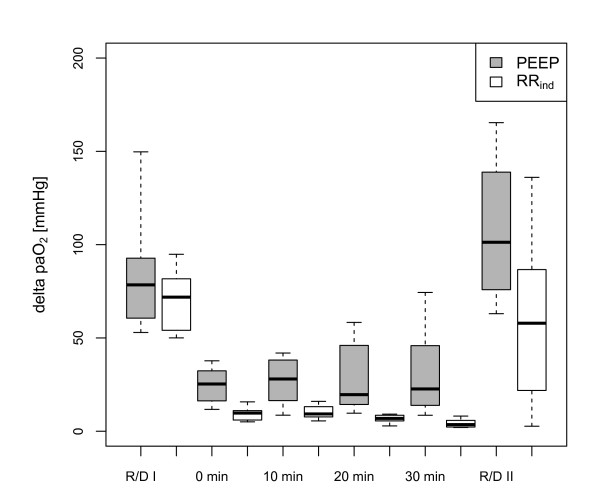
**Time chart of the Δ paO_2_**. Δ paO_2 _(mmHg) after induction (R/D I), within 30 minutes of intervention and after re-induction (R/D II): no respiratory-dependent Δ paO_2 _was founded before provocation. A significantly higher Δ paO_2 _persists following PEEP intervention (p_adjusted _< 0.001 after Bonferroni correction). In four animals of the RR_ind _group no Δ paO_2 _with an amplitude ≥ 50 mmHg was inducible in R/D II due to fixed atelectasis.

**Figure 3:**
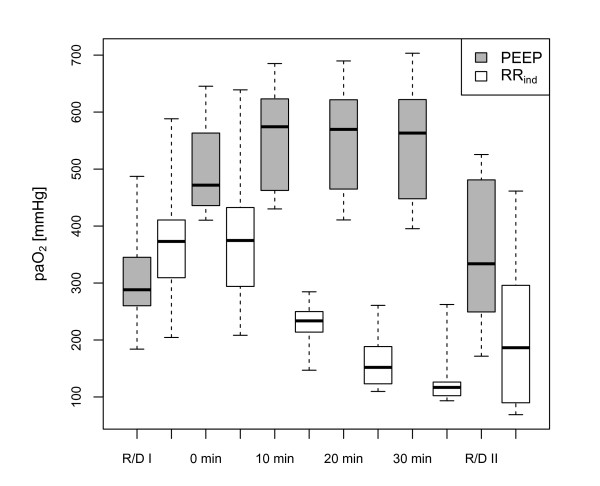
**Time chart of the average paO_2_**. Average paO_2 _(mmHg) after induction (R/D I), within 30 minutes of intervention and after re-induction (R/D II): a significantly decreased oxygenation develops following RR_ind _intervention while PEEP induces a stable lung recruitment (p_adjusted _< 0.001 after Bonferroni correction).

Cyclic R/D measured by respiratory-dependent oscillations of the paO_2 _was provoked in every animal. A median Δ paO_2 _of 78 (IQR = 22) mmHg in the PEEP group and 72 (IQR = 26) mmHg in the RR_ind _group was observed. The corresponding median paO_2 _values were 288 (IQR = 68) mmHg (PEEP group) and 372 (IQR = 94) mmHg (RR_ind _group). These differences were not statistically significant (ΔpaO_2_: *P *= 0.462; paO_2_: *P *= 0.130). As a result of the defined protocol for adjusting RR and PEEP, the RR_ind _intervention consisted of a titration to a RR of 15 ± 1 minute^-1^, while in the PEEP group the PEEP increased up to 13 ± 2 mbar. In both groups an immediate reduction of the Δ paO_2 _was achieved (Figure 2). The Δ paO_2 _reduction was more pronounced in the RR_ind _group while in the PEEP group a higher Δ paO_2 _persisted over 30 minutes (*P*_adjusted _< 0.001 after Bonferroni correction). The application of high PEEP induced an increase in the average paO_2. _In the RR_ind _group the reduction of Δ paO_2 _was in contrast accompanied by a large decrease in average paO_2 _over 30 minutes (Figure 3; *P*_adjusted _< 0.001 after Bonferroni correction). At the end of every experiment the original settings to provoke R/D were repeated to restore R/D (R/D II). In the PEEP group the readjustment to the initial respiratory setting (Table 1) induced a higher Δ paO_2 _(101 (IQR = 59) mmHg) than recorded before the PEEP increase. In the RR_ind _group the Δ paO_2 _was 58 (IQR = 53) mmHg, while in four animals the Δ paO_2 _was < 50 mmHg despite a P_peak _increase up to 45 ± 7 mbar. Hence, the PEEP intervention led a higher amount of recruitable lung tissue, while the RR_ind _induced an increase of non-recruitable, fixed atelectasis.

### Analysis of the ventilation/perfusion distribution

**Figure 4:**
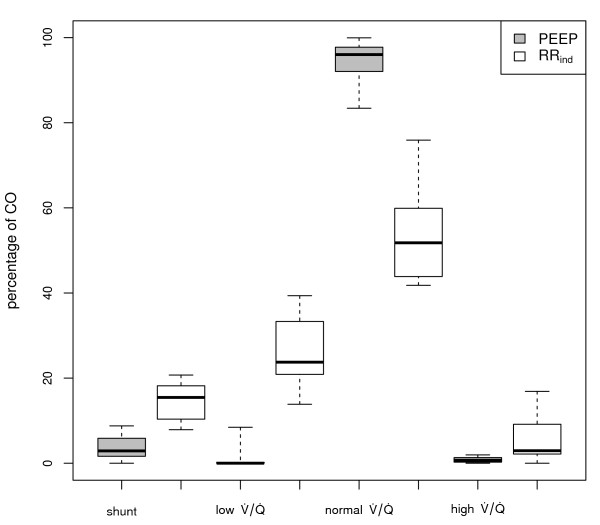
**Ventilation/Perfusion distribution after PEEP or RR_ind _intervention**. MMIMS-MIGET derived $\overset{\cdot }{\text{V}}/\overset{\cdot }{\text{Q}}$ after 30 minutes of intervention: maintained lung recruitment in the PEEP group and impaired pulmonary function in the RR_ind _group. Intergroup differences: shunt, low and normal $\overset{\cdot }{\text{V}}/\overset{\cdot }{\text{Q}}$ each *P *≤ 0.001, high $\overset{\cdot }{\text{V}}/\overset{\cdot }{\text{Q}}$*P *= 0.012 (Mann-Whitney-U-Tests).

The perfusion-based $\overset{\cdot }{\text{V}}/\overset{\cdot }{\text{Q}}$ analysis showed healthy conditions (normal $\overset{\cdot }{\text{V}}/\overset{\cdot }{\text{Q}}$: 96 ± 5% in the PEEP group; 90 ± 14% in the RR_ind _group). A sustained impairment occurred following ALI induction before induction of Δ paO_2 _in both groups (normal $\overset{\cdot }{\text{V}}/\overset{\cdot }{\text{Q}}$: 65 ± 11% versus 74 ± 14%). After 30 minutes of intervention (Figure 4) the PEEP application led to pulmonary recruitment corresponding to the reported paO_2 _values: shunt fraction of 3% (IQR = 4), low $\overset{\cdot }{\text{V}}/\overset{\cdot }{\text{Q}}$ 0% (IQR = 0.2), normal $\overset{\cdot }{\text{V}}/\overset{\cdot }{\text{Q}}$ 96% (IQR = 4) and high $\overset{\cdot }{\text{V}}/\overset{\cdot }{\text{Q}}$ 1% (IQR = 1). Compared to the RR_ind _group (shunt 15% (IQR = 6), low $\overset{\cdot }{\text{V}}/\overset{\cdot }{\text{Q}}$ 24% (IQR = 9), normal $\overset{\cdot }{\text{V}}/\overset{\cdot }{\text{Q}}$ 52% (IQR = 15), high $\overset{\cdot }{\text{V}}/\overset{\cdot }{\text{Q}}$ 3% (IQR = 6)), a considerably improved overall lung function defined by the MMIMS-MIGET was witnessed. Venous admixture was calculated from blood gas data at baseline and after initiation of the respective interventions, but not during presence of high Δ paO_2_, due to high fluctuations of the blood gases (Table 1). 

### Haemodynamics

The haemodynamic parameters are summarised in Table 2. To maintain stable testing conditions despite the challenging ventilator setting in this R/D model, a decrease of the MAP < 60 mm Hg was treated by continuous i.v. administration of epinephrine. The vasopressor requirement to achieve the defined criterion was higher in the PEEP group (*P *< 0.05). No intergroup differences in MAP and CO (each *P *> 0.05) were detected over 30 minutes. The mixed venous oxygen saturation (SvO_2_) decreased over time in the RR_ind _when compared to the PEEP group (*P *< 0.05). 

**Table 2:** Haemodynamic data and vasopressor support (median (IQR))

	PEEP group					RR_ind _group				
Parameter	Baseline ALI	R/D I	Intervention Initial	Intervention 10 to 30 minutes	R/D II	Baseline ALI	R/D I	Intervention Initial	Intervention 10 to 30 minutes	R/D II
MAP (mmHg)	91 (29)	73 (18)	61 (24)	66 (4)	74 (12)*	99 (26)	84 (24)	78 (34)	74 (24)	61 (4)*
CO (l min^-1^)	3.7 (1.1)	3.7 (1.1)		2.9 (0.6)		3.3 (0.7)	3.5 (0.8)		3.1 (1.0)	
SvO_2 _(%)	83 (14)			78 (8)*		81 (8)			66 (12)*	
Epinephrine (μg kg^-1 ^min^-1^)	0	0	0.2 (0.1)*	0.2 (0.1)*	0.2 (0.1)*	0	0	0 (0.1)*	0 (0.02)*	0*

* indicate intergroup difference (*P *< 0.05, Mann-Whitney-U-Test).

## Discussion

The present study compares the effect of an individually titrated RR versus high extrinsic PEEP on respiratory-dependent oscillations of the paO_2 _in a porcine model of cyclic R/D.

### Occurrence of Δ paO_2 _in experimental models

Small amplitudes of Δ paO_2 _ranging from 8.9 to 20 mmHg have been reported in different anaesthetised and mechanically ventilated animal species in a healthy state [10,11,21]. The overall average of Δ paO_2 _amplitude (77 ± 25 mmHg, n = 16) during the initial period of maximal cyclical recruitment (R/D I) is well above this range. In lung lavaged rabbits Δ paO_2 _values of 390 ± 39 mmHg and 283 ± 128 mm Hg were described by Baumgardner *et al. *[11] and Pfeiffer *et al. *[22]. Much lower amplitudes of Δ paO_2 _(estimated at up to 69 mmHg) were reported in a canine lavage model [23]. Few exemplary data of cyclic R/D measured by Δ paO_2 _in porcine models are available [9]. In our model, prolonged exhalation times (8.0 ± 1.4 s, n = 16) produced the largest Δ paO_2_, while in rabbits a 2:1 inverse ratio ventilation (average exhalation time 2.9 s) induced the highest Δ paO_2 _[17]. The occurrence of maximum Δ paO_2 _when exhalation time is longer than inhalation time suggests that, in contrast to rabbit models, derecruitment during exhalation is slower than recruitment during inhalation. Our data suggest that in lavaged pig lungs, minor amounts of Δ paO_2 _occur even at low RR and larger inspiratory and expiratory time intervals are required to induce R/D in large animals.

### Influence of RR and PEEP on Δ paO_2 _

After the demonstration by dynamic computed tomography that collapse of lung tissue is a time-dependent process [15,16], the impact of an increased respiratory rate and associated diminished exhalation time on cyclic R/D was investigated in rabbit models [11,17]. Increased respiratory rate in lavaged rabbits reduced cyclic R/D and maintained end-expiratory recruitment, an effect that was not mediated by intrinsic PEEP. The RR_ind _induced reduction of Δ paO_2 _similarly was not related to a relevant intrinsic PEEP in our study (Table 1). The current study featured a titration of the RR under real-time monitoring of R/D to achieve the targeted Δ paO_2 _and was compared to the maximum PEEP that did not compromise haemodynamic stability. Furthermore, the interventional settings (high PEEP, RR_ind_) were maintained for a longer period of 30 minutes. Both interventions immediately induced a decrease of Δ paO_2_. In the short run, RR_ind _more effectively reduced respiratory-dependent oscillations of the paO_2_, while in the PEEP group a measurable Δ paO_2 _persisted over 30 minutes (Figure 2). 

In contrast to a prior study [17] and despite the favourable effect on Δ paO_2_, the RR_ind _intervention clearly failed to maintain or increase oxygenation like the PEEP application. These findings are reflected in sustained impairment of the $\overset{\cdot }{\text{V}}/\overset{\cdot }{\text{Q}}$ in the RR_ind _group. The MMIMS-MIGET showed a persistent average shunt fraction in both groups (Figure 4), which was consistent with the conventional shunt calculation (Table 1). Neither MMIMS-MIGET shunt nor conventional calculation, however, offers a dynamic assessment of the pulmonary shunt that is capable of following fast, respiratory-dependent variations. The persistence and intergroup differences of Δ paO_2 _are, therefore, not directly related to the average shunt values. The significantly lower amount of atelectasis in the PEEP group (shunt fraction: 3% (IQR = 4)) is still exposed to high peak pressures and long exhalation times and may, therefore, be recruited cyclically. In the high range of paO_2_, even very small changes in shunt fraction around a mean value of 3% are capable of producing substantial Δ paO_2 _(online supplement of [11]). The persisting Δ paO_2 _of 23 (IQR = 26) mmHg in the PEEP group is, therefore, not inconsistent with the small average shunt of about 3%. In contrast, following the RR_ind _intervention the average paO_2 _is much lower and the average shunt fraction is much larger, but the small oscillations of paO_2 _suggest that very little of the atelectatic lung is recruited cyclically. Therefore, there appears to be less cyclic R/D in the RR_ind _group, compared to the PEEP group.

The optimal level of PEEP in ALI is still controversial and presumably depends on the extent and severity of the underlying injury [13]. Referring to the "baby lung" concept, a forced reopening of all collapsed lung areas may lead to further injury and hyperinflation of the lung parenchyma [24,25]. Optimal parameters to titrate the RR remain unclear since the guidance by Δ paO_2 _alone was effective only in diminution of Δ paO_2_, but resulted in a markedly decreased average paO_2 _and lung recruitment. Although the RR_ind _approach reduces the exhalation time per breath cycle, our results suggest that in this model R/D is limited through RR_ind_, not by preventing end-expiratory collapse, but rather by limiting inspiratory recruitment. The RR may play a role as an intervention for cyclic R/D only if gas exchange is maintained by an adequate PEEP.

The slow approach to a steady state average paO_2 _after RR_ind _(over 30 minutes in Figure 3) also supports the assumption that R/D in saline-lavaged pigs is a fairly slow process. Cyclic R/D may not have been completed within a single inspiration or exhalation in this experiment. The initial provocation of cyclic R/D offers comparable findings (Figure 1) in that immediately after R/D induction there was often a pronounced slow drift in the average paO_2_.

### Influence of RR and PEEP on haemodynamics

Especially in a state of compromised haemodynamics, respiratory interventions should avoid negative haemodynamic effects. Syring *et al. *[17] noted a greater CO and SvO_2 _when the RR was used to prevent end-expiratory collapse. Consistent with this concept the animals in the RR_ind _group required less vasopressor administration (*P *< 0.05). The haemodynamic alterations were regarded as only secondary in the present study. The primary focus was set on maintaining stable conditions for the assessment of respiratory-dependent paO_2 _variations. It should be noted that the protocol for vasopressor support was based on MAP. The decreases in MAP in the high PEEP group could be associated with left ventricular afterload reduction rather than compromised CO. The SvO_2 _declined over 30 minutes in the RR_ind _setting despite comparable MAP and CO. However, this may be related to the impaired pulmonary gas exchange and oxygenation rather than an effect of reduced CO.

### Detection of R/D

Cyclic R/D is not detectable by currently available routine clinical monitoring. Dynamic computed tomography, the current gold standard, offers the highest temporal and spatial resolution to monitor dynamic processes like R/D [26]. However, radiation exposure and the need for patient transport limit the practicability in the critically ill. The indirect method focussing on Δ paO_2 _and varying shunt fractions by an indwelling, invasive probe is an experimental technology. The concept of detecting R/D indirectly by Δ paO_2 _may lead to translation to the clinics as related approaches might be developed in the near future [9,27,28].

### Limitations of the study

The lavage model imitates certain features of clinical ALI, particularly surfactant dysfunction and depletion, but, of course, is not identical with ALI. It is widely used to examine the extent and characteristics of atelectasis and alveolar recruitment [29]. Additionally, our experimental model was designed to produce a mild degree of injury with a small amount of fixed shunt and a large amount of recruitable atelectasis. More severe degrees of injury will require further studies. Finally, our observations were collected over a relatively short period of time, compared to the clinical course of ALI. Although some experimental studies indicated a more severe damage and higher inflammatory response in recruiting and derecruiting lung areas [30,31], the long-term extent and impact on human ALI is yet to be determined.

Two experimental technologies were applied in the present study. The MMIMS is a novel and alternate method of MIGET [32,33]. Only the detection of the inert gases by mass spectrometry differs from the conventional gas chromatographic method. The MMIMS technology is still being evaluated in on-going studies. In a prior study, the MMIMS-MIGET derived shunt fraction was shown to be highly correlated to the conventionally calculated shunt [19]. The ultrafast paO_2 _measurement was previously applied and described in detail by several experimental studies [11,17,18,22,30,34]. But a rapidly responding paO_2 _probe is not yet available for clinical use. One limitation, in the clinical setting, for the use of Δ paO_2 _as an index of varying shunt fraction is that the oscillation amplitude is blunted at low haemoglobin saturations. Beyond a paO_2 _of 90 mmHg a complete saturation of the haemoglobin is maintained [35], which allows for direct comparison of average and Δ paO_2 _values.

The peak inspiratory pressure was held on a constant, high level during interventions in both groups, since the influence of the inspiratory pressure on alveolar recruitment is well known [36-38]. Due to the standardisation of the peak pressure following the intervention, however, the tidal volume differed between the two groups, while in the PEEP group also the mean airway pressure increased (Table 1).

## Conclusion

The effects of extrinsic PEEP and a titrated RR on varying shunt fractions through cyclic R/D were characterised in a porcine lavage model. Different kinetics of alveolar recruitment were seen compared to previous small animal models.

Respiratory-dependent oscillations of the paO_2 _were reduced to a higher degree by high respiratory rates than by the feasible PEEP in the present model. The RR titration, however, resulted in a significantly lower average oxygenation and lung recruitment, compared to the use of high PEEP.

## Key messages

• Cyclic alveolar recruitment measured by respiratory-dependent oscillations of the paO_2 _regularly occurs in saline-lavaged pigs during non-protective ventilation.

• As single interventions, increased RR and PEEP both significantly reduce respiratory-dependent oscillations of the paO_2_.

• PEEP maintains end-expiratory recruitment despite persisting amounts of cyclic recruitment, while RR as a single intervention does not maintain sufficient oxygenation and lung recruitment.

• Maximal cyclic R/D in this large animal model is induced by prolonged exhalation times, in contrast to the maximal cyclic R/D by prolonged inspiration reported in a small animal model.

## Abbreviations

ALI: acute lung injury; BE: base excess (mmol l^-1^); CO: cardiac output (l min^-1^); FiO_2: _fraction of inspired oxygen; Hb: haemoglobin (mg dl^-1^) I:E: Inspiration to Expiration ratio; IQR: interquartile range; i.v.: intravenous; MAP: mean arterial pressure (mmHg); MMIMS-MIGET: micropore membrane inlet mass spectrometry - multiple inert gas elimination technique; paO_2_: arterial partial pressure of oxygen (mmHg); paO_2_-Foxy: paO_2 _measured by the FOXY AL-300 probe (mmHg); ΔpaO_2_: respiratory-dependent oscillations of paO_2_; PEEP: positive end-expiratory pressure (mbar); P_endinsp_: end-inspiratory pressure (mbar); P_mean_: mean airway pressure (mbar) P_peak_: peak inspiratory pressure (mbar); Q_s_/Q_t_: calculated pulmonary shunt fraction (%); R/D: cyclic alveolar recruitment and derecruitment; RR: respiratory rate (min^-1^); RR_ind_: individually titrated RR (min^1^); SD: standard deviation; SvO_2_: mixed venous oxygen saturation; T_insp_: inspiration time; T_exp_: expiration time; $\overset{\cdot }{\text{V}}/\overset{\cdot }{\text{Q}}$: ventilation/perfusion distribution; VALI: ventilator-associated lung injury.

## Competing interests

JEB is the owner of Oscillogy LLC, which commercially distributes the MMIMS-MIGET system. JEB participated in set up and design of this study, but had no influence on data acquisition and analysis. None of the other authors declares a conflict of interest.

## Authors' contributions

EKH coordinated and supervised the experiments. EKH, SB, AB, BD and KUK carried out the experiments. EKH, BD and SB performed the data analysis. AE conducted the statistical analysis. EKH drafted the manuscript. KM, MD and JEB, participated in the study design, supervision of the laboratory and revision of the manuscript. All authors edited and approved the final manuscript.
